# Role of Shear Flow on Structure Development during Post-Processing Annealing for Poly(lactic acid)

**DOI:** 10.3390/polym15030693

**Published:** 2023-01-30

**Authors:** Hoang-Giang Dai Vo, Takumitsu Kida, Masayuki Yamaguchi

**Affiliations:** 1 School of Materials Science, Japan Advanced Institute of Science and Technology, 1-1 Asahidai, Nomi 923-1292, Ishikawa, Japan; 2 Research Center for Carbon Neutral, Japan Advanced Institute of Science and Technology, 1-1 Asahidai, Nomi 923-1292, Ishikawa, Japan

**Keywords:** poly(lactic acid), molecular orientation, crystallization, annealing

## Abstract

The effect of shear history on structure development during post-processing annealing was studied using poly(lactic acid) PLA. Since PLA shows a low crystallization rate, quenched films had no crystallinity. Moreover, molecular orientation was not detected in the films. During the annealing procedure beyond its glass transition temperature, however, molecular orientation to the flow direction occurred with the crystallization growth in the films having an appropriate shear history. This peculiar crystal growth during the annealing was most probably attributed to the crystallization from extended chain crystals generated during the applied shear history, although the amount of extended chain crystals was low. The results obtained in this study should be noted because the molecular orientation proceeded due to the annealing history applied. Furthermore, this phenomenon will be used to suppress dimensional change and increase product rigidity.

## 1. Introduction

Poly(lactic acid) (PLA), as a biomass-based eco-friendly plastic, is expected to be employed instead of conventional petroleum-based plastics due to its high rigidity and high melting point [1,2,3,4,5,6], although some deficits including mechanical brittleness and poor processability have to be solved. Up to now, therefore, various modification techniques, including blending and processing operations, have been proposed [1]. For further applications of PLA, the enhancement of crystallinity must be very important. Once the crystallinity is high, the heat resistances of PLA, such as heat deflection temperature, Vicat softening temperature, and ball pressure temperature, are comparable with those of isotactic polypropylene, and better than those of poly(vinyl chloride), polystyrene, polyethylene, and poly(methyl methacrylate) [1,2,3,4,5,6]. Considering that the rigidity of PLA is higher than those of the conventional plastics, it is quite attractive. Therefore, the market of PLA is increasing greatly recently.

Because PLA shows a low lateral growth rate (or linear growth rate) at crystallization, the crystallinity of a product obtained by a conventional processing method is usually very low. Consequently, its glass transition temperature *T_g_*, which is around 58 °C [1,2,3,4,7], decides the heat resistance of a product, which prohibits its wide applications. From the viewpoints of the processing operation, cooling at an appropriate temperature is important to enhance the crystallization rate. It has been known that PLA shows the highest lateral growth rate at 130 °C as a crystallization temperature, which is the medium temperature between its equilibrium melting point 207 °C [8] and *T_g_*. However, even at 130 °C, the crystallization rate is still very low compared to conventional crystalline plastics. Furthermore, water cannot be used as a cooling medium at 130 °C under atmospheric pressure, which results in the detriment of cost performance. The appropriate crystallization temperature showing the highest lateral growth rate can be lowered by the addition of a plasticizer, which is one technique to increase the crystallization rate using water. Moreover, the maximum lateral growth rate is known to increase by the plasticization owing to the enhancement of chain mobility [1,9,10,11]. Up to now, polyester compounds with low molecular weight, poly(ethylene glycol), oligomeric PLA, glycerol, cardanol and its derivatives, and citrate esters have been studied intensively and commonly employed in industry.

Another well-known technique to increase the crystallization rate is the addition of crystal nucleating agents, such as carbon nanotube [12], talc [12], calcium carbonate [12], boron nitrade [12], orotic acid [12,13,14], zinc phenylphosphonate [14,15], hydrazide compounds [14,16], and amide compounds [12,14,17,18]. Of course, this technique can be used with the addition of a plasticizer. Under a quench process, however, the crystallinity of an obtained product containing a nucleating agent and/or a plasticizer is not high enough to show good heat resistance. Therefore, post-processing annealing is often carried out in industry [19,20,21,22,23]. In general, exposure to annealing operation beyond *T_g_* leads to high crystallinity with reduced molecular orientation. As a result, the dimension of a product usually varies, e.g., shrinkage in the flow direction [24,25,26]. In fact, it was reported that the shrinkage was detected in PLA products by post-processing annealing operation [19,21,22,23]. This may be a serious problem for products employed with other materials, such as metals and ceramics.

To increase the crystallinity, flow-induced crystallization is also effective for polymers, which is applicable to PLA [27,28,29,30,31,32]. Under flow field, chain stretching and/or orientation occurs, which can be predicted by the Weissenberg number *Wi*. It is known that *Wi* is defined by the product of the characteristic time *τ* for a specific relaxation mode and the strain rate $\dot{\gamma }$ as follows;

(1) $$Wi\equiv \tau \dot{\gamma }$$

When the reptation motion is considered as a relaxation mode, *Wi* provides the information on molecular orientation during flow. In this case, molecular orientation, and therefore, residual stress, in a product is pronounced when an applied strain rate is larger than the inverse of reptation time, i.e., *Wi* > 1. Once *τ* is the Rouse relaxation time, *Wi* gives the information on chain stretching. The extended chain crystals are formed when an applied strain rate is larger than the inverse of Rouse relaxation time. Since the Rouse relaxation time is proportional to the square of the molecular weight, a polymer containing a high-molecular-weight fraction tends to form a shish-kebab structure, in which the highmolecular-weight fraction forms shish, i.e., extended chain crystal. As a result, its crystallization occurs in a short time, even though the diffusion constant becomes small with increasing the molecular weight. Of course, products obtained show high crystallinity as well as high molecular orientation, giving the high modulus and strength as well as good heat resistance because of high crystallinity. Besides the molecular weight, the processing temperature is also important because it decides the relaxation time. The effect of the processing temperature can be predicted by the time-temperature superposition principle. Near the glass transition temperature, the well-known WLF equation predicts the temperature dependence of rheological parameters including the relaxation time. When the processing temperature is 50 °C higher than the glass transition temperature, an apparent flow activation energy, obtained by the Andrade equation, predicts the temperature dependence. Of course, extended chain crystals form under a lower strain rate at a low temperature because the Rouse time increases.

In this study, the effect of applied flow history on the crystallization and chain orientation during post-processing annealing was investigated considering *Wi* during the shear history. Since the rigidity and heat resistance of a product are strongly affected by the crystallinity and chain orientation, the results obtained here must provide important information on the processing technique to improve the performance of PLA products in industry. Moreover, a similar phenomenon will be detected in other crystalline polymers showing slow crystallization. Therefore, the present study may establish a new method of material design from the viewpoint of processing operation.

## 2. Experimental

### 2.1. Material

A commercially available PLA (Ingeo 4032D; NatureWorks, Minnetonka, MN, USA) with 1.5% D-isomer was used in this work. The density at room temperature is 1240 kg/m^3^, and the melting point is 167 °C. The number- and weight-average molecular weights, evaluated by size-exclusion chromatography (HLC-8020; Tosoh, Tokyo, Japan) with standard polystyrene samples, were 1.0 × 10^5^ and 1.8 × 10^5^, respectively.

### 2.2. Sample Preparation

After PLA pellets were dried under vacuum for 4 h at 80 °C, compression molding was performed at 200 °C under 10 MPa for 2 min to prepare flat sheets with a thickness of 1.2 mm. They were subsequently quenched by a cooling unit at 25 °C under 10 MPa for 5 min. The sheets obtained were kept in a vacuum chamber at room temperature to prevent moisture before use.

**Figure 1:**
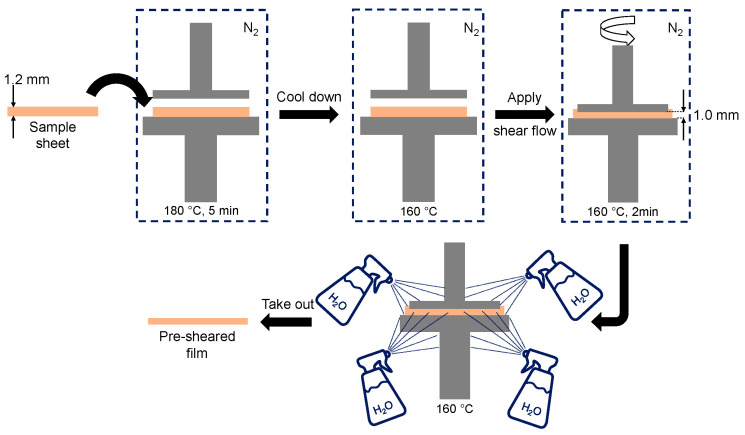
Illustration of the sample preparation method.

The sample sheet was set in a parallel-plate rheometer with 25 mm diameter (MR500; UBM, Mukō, Japan) at 180 °C for 5 min to melt the sample. After a temperature jump from 180 °C to 160 °C, a shear flow was applied for 2 min. The shear rate at the edge of the plate was 30 s^−1^. The distance between the plates was 1 mm. After the cessation of shear flow, the sample was immediately quenched by spraying water and taken out after solidification. The procedure is shown in Figure 1.

The obtained film was cut into two parts by a razor blade. One of them was annealed at 80 °C for various periods using a temperature controller (Mettler FP90; Mettler Toledo, Columbus, OH, USA), whereas the other was kept in a vacuum chamber for further characterization. Because the edge part was not uniform due to the water spray, we characterized the structure at 10 mm from the center, at which the applied shear rate was 24 s^−1^.

### 2.3. Measurements

The thermal properties were evaluated by a differential scanning calorimeter (DSC; DSC8500; PerkinElmer, Waltham, MA, USA) under a purified nitrogen flow. Approximately 7.5 mg of the sample was placed in an aluminum pan. The sample was heated from 30 °C to 200 °C at 30 °C/min. Then, the crystallization behavior was evaluated at a cooling rate of 1 °C/min.

The frequency dependence of the oscillatory shear modulus was evaluated by a cone-and-plate rheometer (AR2000ex, TA Instruments, New Castle, DE, USA) at 160 °C, 170 °C, 180 °C, 190 °C, and 200 °C. The radius of the plates was 25 mm, and the cone angle was 4 °. The angular frequency was swept from 629.3 to 0.01 rad/s, and the applied shear strain was within the linear viscoelastic range. All measurements were performed under a nitrogen atmosphere to prevent thermal degradation.

The temperature dependence of the dynamic tensile modulus was evaluated by a dynamic mechanical analyzer (E-4000; UBM) at 10 Hz from room temperature at a heating rate of 2 °C/min. The applied tensile strain was within the linear viscoelastic range. For the measurement of films with shear history in the rheometer, the direction of the oscillatory strain coincided with the flow direction.

The two-dimensional wide-angle X-ray diffraction (2D-WAXD) pattern was collected using an X-ray diffractometer (SmartLab; Rigaku, Akishima, Japan). The CuK𝛼 radiation beam, generated at 45 kV and 200 mA, was inserted in the normal direction at 10 mm from the center of the sample films taken out from the rheometer. The crystallinity was calculated using a fitting software, named “IgorPro”. The Gaussian method with a linear baseline was used to determine the crystallinity.

Molecular orientation was evaluated by a polarized optical microscope (DMLP; Leica Microsystems, Wetzlar, Germany) under crossed polarizers. Optical retardation values were measured five times using a Berek compensator.

## 3. Results and Discussion

### 3.1. Thermal and Rheological Properties of the Sample

**Figure 2:**
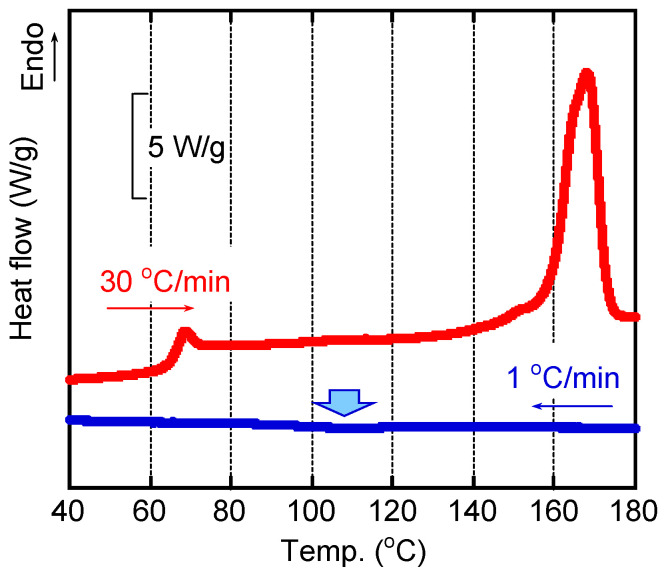
DSC cooling curve at 1 °C/min from 200 °C and the second heating curve at 30 °C/min.

Figure 2 shows the DSC curves of the sample. The blue curve was the cooling one at 1 °C/min. The measurement was started from 200 °C. A weak exothermic peak due to crystallization was detected at around 110 °C as indicated by the bold arrow in the figure. When the cooling rate was 3 °C/min or higher, however, no peak was detected (but not present here). This is reasonable because the crystallization rate of PLA is low as mentioned in the introduction. After cooling to 30 °C at 1 °C/min, the sample was heated again at 30 °C/min, which was denoted by a red line. The melting peak was clearly detected at 167 °C. The crystallinity was calculated from the heat of fusion Δ*h_F_*, by Equation (2), and found to be 30%.

(2) $$\chi_{w}=\frac{\Delta h_{F}}{\Delta h_{F}^{0}}$$

where Δ*h*^0^*_F_* is the heat of fusion for the perfect crystal, 93.1 J/g [33].

**Figure 3:**
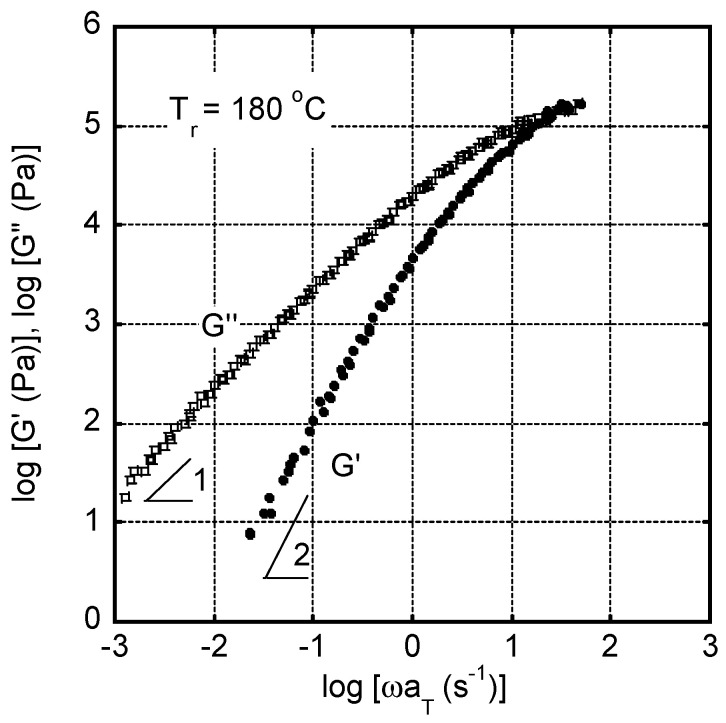
Master curves of shear storage modulus *G′* and loss modulus *G″* as a function of angular frequency *ω* at the reference temperature *T_r_* of 180 °C.

The master curves of angular frequency *ω* dependence of oscillatory shear moduli, such as storage modulus *G’* and loss modulus *G”*, were shown in Figure 3. The reference temperature *T_r_* was 180 °C. Since the measurements were performed from high angular frequency, it took about one hour for the measurement at the lowest angular frequency, i.e., 0.01 s^−1^, at each temperature. As seen in the figure, the time-temperature superposition principle was applicable only by horizontal shifts in this temperature range, suggesting that crystallization did not occur during measurements even at 160 °C, i.e., the lowest temperature for the measurements, although it was lower than the melting point. The apparent flow activation energy was calculated from the shift factors *a_T_* following the Arrhenius-type Andrade equation [34,35], and found to be 90 kJ/mol. The modulus at the cross point, i.e., G’ = G”, was ca. 0.2 MPa, which was much lower than the rubbery plateau modulus, i.e., 1.0 MPa [4]. This was attributed to the broad distribution of relaxation time (molecular weight).

In Figure 2, the rheological terminal region was clearly detected: i.e., *Gʹ* and *G″* were proportional to *ω*^2^ and *ω*, respectively. Therefore, zero-shear viscosity $\eta_{0}$ and steady-state shear compliance $J_{e}^{0}$, as basic rheological parameters in the terminal region, were calculated by the following equations [34], and found to be $\eta_{0}$ = 1.96 × 10^4^ Pa s and $J_{e}^{0}$ = 1.00 × 10^−5^ Pa^−1^ at 180 °C:

(3) $$\eta_{0}=\underset{\omega \to 0}{\lim}\frac{G\prime \prime }{\omega }$$

(4) $$J_{e}^{0}=\underset{\omega \to 0}{\lim}\frac{G^{\prime }}{{G^{\prime \prime }}^{2}}$$

Moreover, the weight-average relaxation time *τ_w_*, defined by Equation (5), was found to be 0.196 s at 180 °C.

(5) $$\tau_{w}\equiv \frac{\int \tau^{2}H\left(\tau \right)d\ln\tau }{\int \tau H\left(\tau \right)d\ln\tau }=\eta_{0}J_{e}^{0}$$

Using the apparent flow activation energy, *τ_w_* at a different temperature can be calculated, e.g., 0.56 s at 160 °C. Therefore, *Wi* associated with the reptation motion at 160 °C should be larger than one at 24 s^−1^, i.e., applied shear rate before quenching. The result indicated that the molecular orientation progressed during the shear flow applied before quenching.

As well known, $\eta_{0}$ is proportional to *M_w_*^3.4^, where *M_w_* is the weight-average molecular weight. According to Dorgan [4], Equation (6) expresses the value of $\eta_{0}$ at 180 °C using the absolute value of *M_w_* of PLA.

(6) $$\log\eta_{0}=-14.26+3.4\log M_{w}$$

The absolute value of *M_w_*, calculated by the equation (not polystyrene-equivalent value), was 286,000 for the present sample. The value is much larger than the critical molecular weight of PLA, which was reported to be 9000–16,000 [1,4].

### 3.2. Effect of Shear History

**Figure 4:**
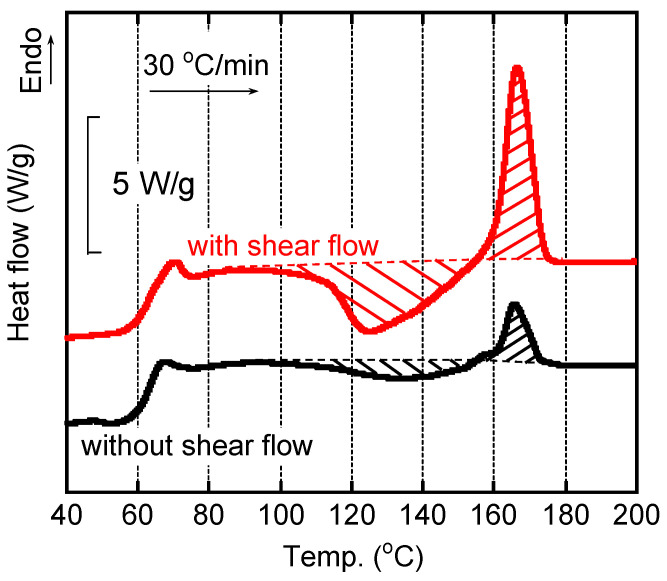
DSC heating curves of the quenched films with/without shear history. The heating rate was 30 °C/min.

The sample films quenched in the parallel-plate rheometer were taken out to evaluate the structure. One sample had the shear history at 24 s^−1^ for 2 min at 160 °C before quenching. The other sample was obtained at the same method, i.e., annealed in the rheometer, but without shear history. At first, we performed the measurements several times and confirmed the reproducibility of the DSC curves shown in Figure 4. It demonstrated that the quench method was good enough to solidify the sample film immediately without crystallization.

As seen in the figure, the DSC heating curves for the quenched films were affected by applied shear history in the rheometer. The film with shear history showed a clear exothermic peak due to cold crystallization, which started from 90 °C. Then, the peak of cold crystallization was located at 124 °C. In contrast, a weak peak was detected at a higher temperature, around 135 °C, for the film without shear history, which had the same thermal history in the rheometer. Because the cold crystallization did not progress sufficiently, the melting peak was weak for the film without shear. For each film, moreover, the heat of fusion for melting was almost identical to that for cold crystallization. The result demonstrated that the quenched films had no/few crystallinities, irrespective of the shear history. The enhancement of cold crystallization for the sheared film was most probably attributed to tiny crystals that were formed during shear, as discussed later.

**Figure 5:**
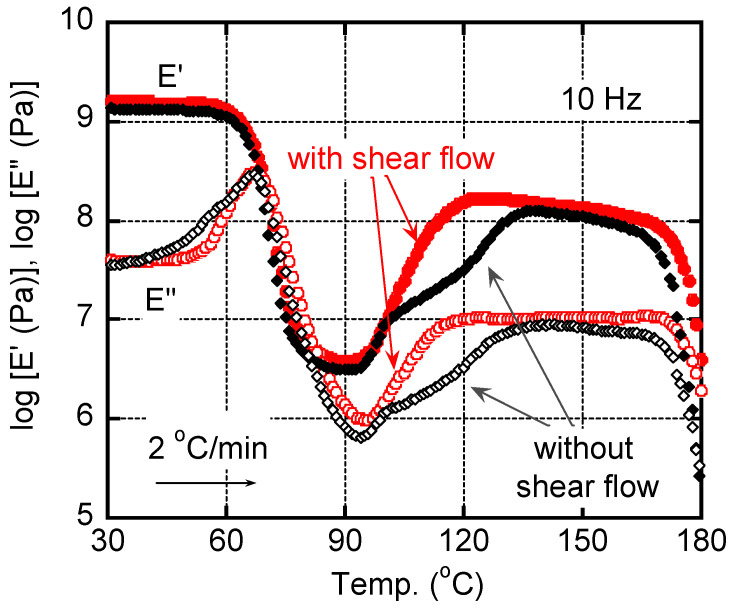
Temperature dependences of tensile storage modulus *E′* and loss modulus *E″* at 10 Hz for the quenched films with/without shear history. The heating rate was 2 °C/min.

Figure 5 shows the temperature dependences of dynamic tensile moduli, such as storage modulus *E′* and loss modulus *E″*, for the quenched films with/without shear history. As seen in the figure, *E′* showed a significant drop in the temperature range from 60 °C to 80 °C, at which *E″* showed a sharp peak ascribed to the glass-to-rubber transition at around 67 °C. The value corresponded with those reported in previous papers [9,11,18,21]. Moreover, the *E′* values increased with the temperature from 90 °C to 120 °C, which was attributed to the cold crystallization. The modulus increase occurred earlier for the film with applied shear history, which corresponded with the DSC result in Figure 4. Although the effect of shear history was not so obvious in the dynamic mechanical properties, it would be owing to the slow heating rate at the measurements, i.e., 2 °C/min for the dynamic mechanical analysis (30 °C/min for the DSC measurements). After the cold crystallization, *E′* became higher than 100 MPa. The values are comparable to those of pure isotactic polypropylene in the same temperature range. In other words, PLA shows a similar rigidity to isotactic polypropylene, when crystallized sufficiently. Beyond 165 °C, both moduli decreased due to melting, at which it occurred at a slightly higher temperature for the film with shear flow. The melting point was also similar to that of isotactic polypropylene.

### 3.3. Structure Development during Annealing

The film with shear history applied in the parallel-plate rheometer, i.e., 24 s^−1^ for 2 min at 160 °C, before quenching, was exposed to annealing at 80 °C in the temperature-controller for various periods. Then, various characterizations were carried out.

**Figure 6:**
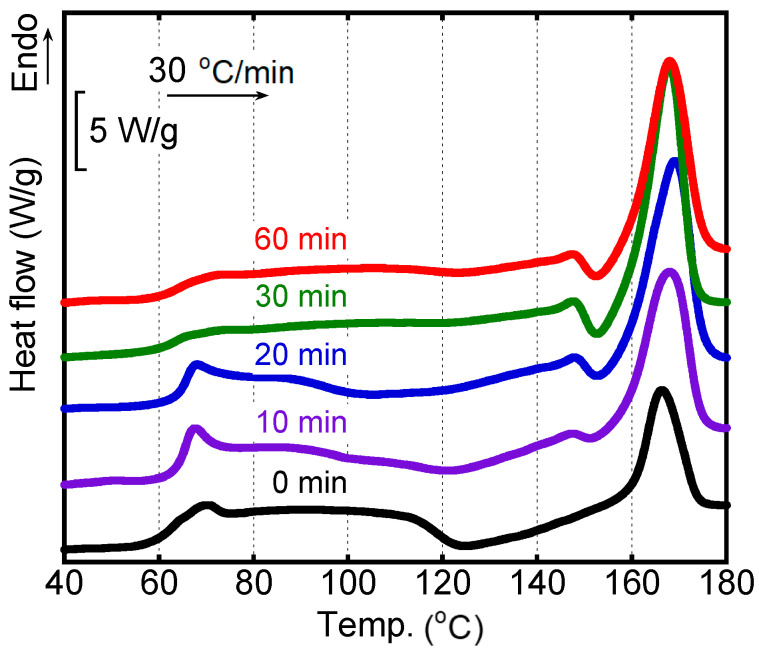
DSC heating curves of the sheared films after annealing at 80 °C for various periods. The heating rate was 30 °C/min.

Figure 6 shows the DSC heating curves of the sheared films after annealing at 80 °C. The heating rate was 30 °C/min. The numerals in the figure denote the annealing periods. The peak area of cold crystallization decreased with the annealing period and was not detected clearly after annealing for 30 min or more. Compared with the film without annealing history, cold crystallization was detected from a low temperature for the films annealed for 10 min and 20 min. Moreover, these samples showed larger peak areas ascribed to melting at around 167 °C. The results suggested that the crystallinity was enhanced by exposure to annealing at 80 °C. Correspondingly, the *T_g_* became obscure owing to the decrease in the amorphous region. Moreover, it was found that prolonged annealing for more than 30 min did not affect the crystallinity greatly.

**Figure 7:**
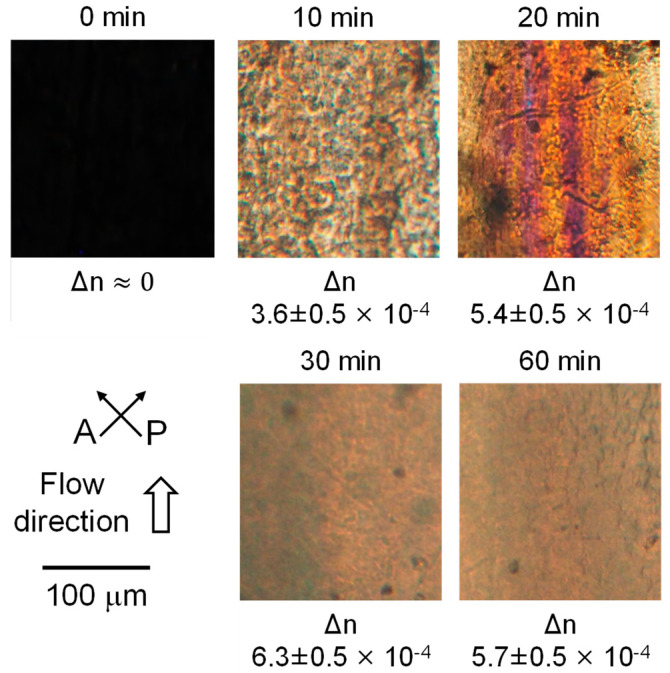
Polarized optical microscope images of the sheared films after annealing at 80 °C for various periods. The annealing periods were denoted at the top of the pictures, and the birefringence values were at the bottom of the pictures.

Figure 7 shows the optical microscope images under crossed polarizers. The directions of the polarizer (P) and analyzer (A) and applied flow in the rheometer were described in the figure. The films having the shear history were exposed to annealing at 80 °C for various periods. The annealing periods were indicated at the top of the pictures.

Although there was no light transmittance through the film without annealing (0 min), indicating no orientation, birefringence color was clearly detected after annealing at 80 °C. The result demonstrated that the molecular orientation progressed during annealing. The optical retardation *Γ* was evaluated using a Berek compensator. Then, the birefringence Δ*n* values were calculated using the film thickness *d* measured by a micrometer, as shown in Equation (7). The results were denoted at the bottom of pictures. As seen in the figure, the birefringence was too small to be measured for the film without annealing (0 min). However, birefringence increased with the annealing period up to 30 min. Since the intrinsic birefringence of PLA is reported to be 0.03 [36], the Hermans orientation function *F*, which was calculated by dividing the measured birefringence Δ*n* by the intrinsic birefringence Δ*n*^0^ as denoted in Equation (8), was found to be ca. 0.021 after annealing for 30 min.

(7) Δn=Γ/d

(8) $$F\equiv \frac{3\langle \cos^{2}\varphi \rangle -1}{2}=\Delta n/\Delta n^{0}$$

where *ϕ* is the angle that a segment makes with the flow direction.

To confirm the orientation, moreover, 2D-WAXD measurements were performed using the sheared films with/without the annealing procedure at 80 °C. The X-ray beam was inserted at 10 mm from the center in the normal direction to the films. It is well known that conventional processing operations usually provide orthorhombic α-form crystals for PLA, which has the following unit cell parameter, *a* = 1.07 nm, *b* = 0.62 nm, and *c* (chain axis) = 1.89 nm, and *γ* = 90 ° [4].

**Figure 8:**
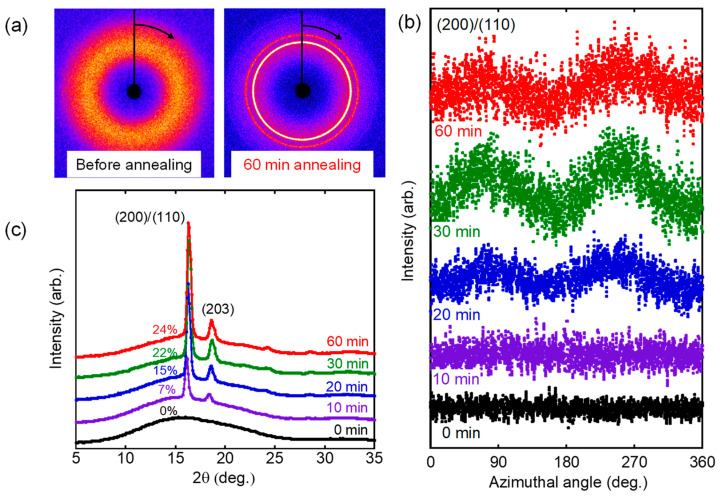
(**a**) 2D-WAXD patterns, (**b**) azimuthal distribution of (200)/(110) plane, and (**c**) 2*θ* profiles of the sheared films after annealing at 80 °C for various periods.

Although it was difficult to find the orientation from 2D-WAXD images directly (Figure 8a), the azimuthal intensity distribution of the strongest diffraction, which was ascribed to the (200)/(110) plane of α-form crystals, clearly showed the peaks on the equator, i.e., at 90 and 270 degrees, after annealing (Figure 8b). In contrast, there was no peak for the film without annealing. The results demonstrated that crystalline chains oriented to the flow direction by the annealing operation, i.e., molecular orientation was provided by the annealing. The orientation increased with the annealing period and became constant beyond 30 min. The result corresponded with the birefringence values in Figure 7. Since 2D-WAXD images in Figure 8a indicated that the crystallinity was enhanced by annealing, the integrated 2*θ* profiles were also plotted as shown in Figure 8c. It was revealed that only a broad halo was detected in the film without annealing (0 min). In contrast, distinct peaks were detected at 16° and 18°, which were ascribed to the (200)/(110) and (203) planes of 𝛼-form crystals, respectively [4,37]. The crystallinity calculated by the following relation *χ_WAXD_* was denoted in the figure.

(9) $$\chi_{WAXD}=\underset{i}{\sum }I_{Ac_{i}}/\left(\underset{i}{\sum }I_{Ac_{i}}+I_{Aa}\right)$$

where *I_ci_* and *I_a_* are the integrated areas of the crystalline and amorphous peaks, respectively.

It was clearly found that the crystallization progressed until the annealing period reached 30 min.

The increase in the molecular orientation during annealing must be attributed to the crystal growth from the extended chain crystals, i.e., shish, which were formed during shearing in the rheometer. Since the amount of shish was low, the crystallinity was not detected by the WAXD and DSC measurements before annealing in this study. Moreover, both WAXD and optical microscope observation did not detect the orientation by the same reason. These crystals formed during shearing showed good nucleation ability during annealing, which was indicated as an enhanced cold crystallization in the DSC heating curve. Moreover, the alignment of shish to the flow direction led to the orientation of crystalline chains grown during annealing. Similar results were recently reported for isotactic polypropylene containing fibrous nucleating agents and polyhydroxyalcanoate [38,39,40], although the crystal growth did not occur during post-processing annealing in these studies. This anomalous growth of molecular orientation for PLA will be important for the dimensional control and mechanical properties, such as modulus and its anisotropy. 

**Figure 9:**
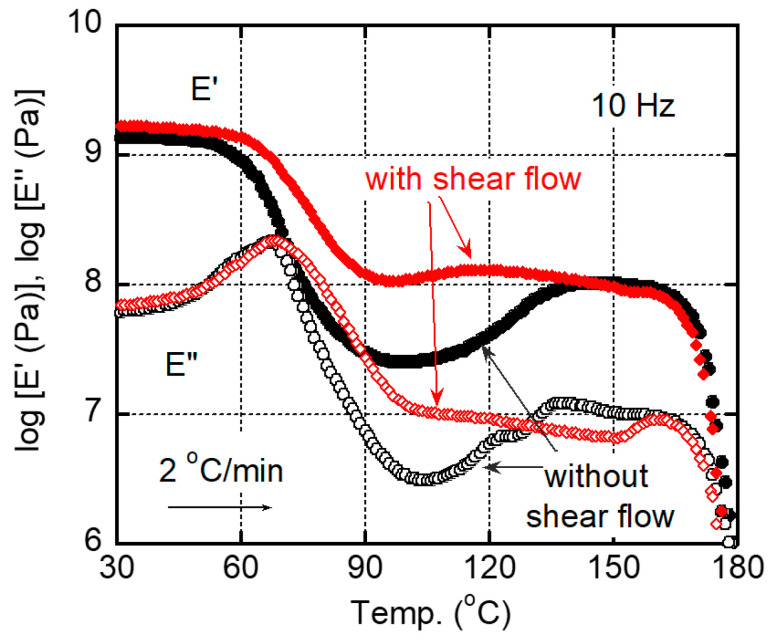
Temperature dependences of tensile storage modulus *E′* and loss modulus *E″* at 10 Hz for the films with/without shear history after annealing at 80 °C for 60 min. The heating rate was 2 °C/min.

Figure 9 shows the temperature dependences of dynamic tensile moduli for the films after annealing at 80 °C for 60 min. One sample had the shear history, and the other did not. It was clear that the film having the shear history showed higher *E’* in the wide temperature region, which was attributed to its high crystallinity with chain orientation. In contrast, the film without the shear history showed the modulus increase beyond *T_g_* clearly due to cold crystallization even after the annealing procedure. The result also demonstrated that the applied shear flow played a key role in reducing the annealing period required for sufficient crystallinity.

Considering that post-processing annealing is an important technique for PLA in industry to enhance the crystallinity and thus the heat resistance, this information must be noted. The control of the primary molecular structure, such as molecular weight distribution and L-lactide content, will be effective to exhibit this anomalous phenomenon more clearly. The broadening of molecular weight is one method to observe this phenomenon, because a high-molecular-weight fraction forms shish even under a low shear rate. PLA comprising only L-lactide will be effective to produce shish. Furthermore, a similar phenomenon should be detected for another crystalline plastic, especially a polymer with a low lateral growth rate at crystallization during cooling.

## 4. Conclusions

Structure development during post-processing annealing, which is an important technique for PLA in industry, was investigated using a quenched PLA film having shear history. It was confirmed that the shear history applied in this study, i.e., 24 s^−1^ for 2 min at 160 °C, did not give the chain orientation at all after quenching, although *Wi* associated with molecular orientation, i.e., reptation, was beyond one. This must be originated from the prompt relaxation of chain orientation before solidification, i.e., glassification. Furthermore, crystallinity was not detected by DSC and WAXD after quenching. However, tiny amounts of extended chain crystals must exist while keeping their orientation to the flow direction to some degree. During the post-processing annealing operation beyond *T_g_*, these crystals acted as nuclei, which was indicated by accelerated cold crystallization in DSC measurements. In addition, the azimuth angle distribution of a crystal diffraction peak obtained by 2D-WAXD measurements revealed that the chain orientation of the crystalline phase progressed. Of course, annealing increased the orientation birefringence, as confirmed by the polarized optical microscope observation. The present results are expected to provide important information for improving dimensional control, rigidity and its anisotropy, and heat resistance after annealing. Utilizing this phenomenon, PLA will be used in various applications, including injection-molded products, such as automobile parts and electronics housing parts. In particular, with the recent development of electric vehicles, there is a strong demand for lighter weight automobile parts. Increased rigidity allows the thickness of automobile parts to be reduced, leading to reduction of weight. Additionally, dimensional control is an advantage employed in other material combinations, such as metals and ceramics, which are main parts of automobile applications. Of course, the development of flexible packaging prepared by film processing, including T-die extrusion and tubular-blown method, is another important target for PLA as awareness of environmental issue increases greatly these days. For the film applications, rigidity must be very important to decide the thickness. Of course, the heat resistance should be greatly improved by this technique, i.e., annealing after appropriate shear history.

## Data Availability

Not applicable.
